# Implication of β2-adrenergic receptor and miR-196a correlation in neurite outgrowth of LNCaP prostate cancer cells

**DOI:** 10.1371/journal.pone.0253828

**Published:** 2021-06-30

**Authors:** Ilaria Guerriero, Håkon Ramberg, Krizia Sagini, Manuel Ramirez-Garrastacho, Kristin A. Taskén, Alicia Llorente

**Affiliations:** 1 Department of Molecular Cell Biology, Institute for Cancer Research, Oslo University Hospital, Oslo, Norway; 2 Biogem, Istituto di Biologia e Genetica Molecolare, Ariano Irpino, Avellino, Italy; 3 Department of Tumor Biology, Institute for Cancer Research, Oslo University Hospital, Oslo, Norway; 4 Institute of Clinical Medicine, University of Oslo, Oslo, Norway; 5 Department of Mechanical, Electronics and Chemical Engineering, Faculty of Technology, Art and Design, Oslo Metropolitan University, Oslo, Norway; University of Catanzaro, ITALY

## Abstract

The β2-adrenergic receptor has been shown to be involved in neuroendocrine differentiation and to contribute to the development of aggressive prostate cancer. In this study we have investigated whether miR-196a plays a role in the regulation of the β2-adrenergic receptor in the LNCaP prostate cancer cell line. Our results show that the expression of miR-196a is elevated in LNCaP prostate cancer cells with reduced levels of β2-adrenergic receptor after stably transfection with three different shRNAs. Furthermore, treatment with β-blockers showed that this upregulation is strictly related to the low levels of β2-adrenergic receptor and not to the inhibition of the receptor signaling activity. Finally, we found that the reduced ability of LNCaP cells with low levels of β2-adrenergic receptor to initiate neuroendocrine differentiation under androgen depletion conditions is mediated by miR-196a. In conclusion, this study provides the rational for a role of miR-196a in the β2-adrenergic receptor mediated neuroendocrine differentiation of LNCaP prostate cancer cells.

## Introduction

Prostate cancer (PCa) was the second most commonly diagnosed cancer among males worldwide in 2018 (13.5% of new cancer cases) [1]. Furthermore, the disease caused over 350,000 deaths (6,7% of cancer-related male deaths) [1]. Curative therapy of prostate cancer often involves radical treatment such as prostatectomy or radiotherapy. However, 27%-53% of patients undergoing these therapies will develop biochemical recurrence (BCR), which is considered to be predictive of the development of metastases and clinical recurrence [2, 3]. When the disease has disseminated to other tissues, patients are commonly treated with androgen deprivation therapy. This therapy targets the androgen receptor (AR), a receptor that plays a crucial role both in the physiology of the prostate and pathogenesis of prostate cancer [4]. Several therapeutic molecules targeting the AR signaling have been developed, such as abiraterone, enzalutamide and apalutamide, but the response is often transient and patients eventually develop castration-resistant prostate cancer (CRPC), which is an incurable disease [5, 6].

The development of CRPC involves different mechanisms that are not fully understood [7]. A mechanism that has been investigated is signaling through the β2-adrenergic receptor (ADRB2) [8]. ADRB2 is a member of the G-protein-coupled receptor superfamily and, interestingly, both the ADRB2 mRNA and protein levels are generally increased in PCa cells compared to benign prostate cells [9–11]. In addition, the signaling pathway of ADRB2 through activation of adenylyl cyclase, cAMP production and activation of protein kinase A has been shown to be involved in neuroendocrine differentiation (NED), a process that has been associated with disease progression and poor prognosis [12–16]. However, chronic ADRB2 activation causes a reduction in the levels of ADRB2, resulting in de-differentiation and epithelial-mesenchymal transition (EMT) [17].

MicroRNAs (miRNAs) are small non-coding single strand RNAs of 19–22 nucleotides. These small RNAs negatively regulate gene expression at a post-transcriptional level by binding the 3’UTR of target mRNAs and inhibiting their translation into proteins [18]. miRNAs play multiple roles in cancer, and it is well known that the same miRNA can behave as an oncogene or as a tumor suppressor in different pathological contexts by acting on different targets [19]. Interestingly, several miRNAs have been shown to be crucial mediators in PCa progression (miR-18a, miR-106/miR-25, miR-125b), metastasis (miR-21, miR-141, miR-375) and therapy-resistance (miR-32, miR-95, miR-106b) [20, 21]. miR-196a has been shown to be implicated in several cancers, such as pancreatic, breast, colorectal, esophageal and lung carcinomas, as well as leukemias, by promoting cell proliferation, migration, invasion and radiotherapy resistance through several gene targets [22–24]. Moreover, miR-196a has been shown to target more tumor suppressors than oncogenes [24]. The role of this miRNA in PCa has been poorly investigated, but some studies indicate that it is upregulated in PCa compared to normal prostate tissue [25, 26]. Furthermore, we have recently shown that this miRNA is found at lower levels in urinary extracellular vesicles of PCa patients compared to healthy controls [27]. Finally, it has recently been shown that miR-196a stimulates prostate cancer proliferation via downregulation of p27^kip1^, a protein involved in cell cycle regulation [26]. In this study we have investigated the potential role of miR-196a in the context of ADRB2 expression and NED in prostate cancer LNCaP cells.

## Materials and methods

### Cell culture and treatments

The human prostate cancer cell line LNCaP was purchased from the American Type Culture Collection (Rockville, MD) and maintained in RPMI 1640 (cat. no. R0883; Sigma; St. Louis, MO) with 10% fetal calf serum (FCS) (cat. no. F7524; Sigma, St. Louis, MO), 100 units/ml penicillin and 50 mg/ml streptomycin (cat. no. P4458; Sigma, St. Louis, MO), and 1% alanyl-glutamine (cat. no. G8541; Sigma, St. Louis, MO) at 37°C in 5% CO_2_ and humidified air. In experiments involving androgen depletion, phenol red-free medium was used, and FCS was replaced with 10% charcoal-stripped serum (CSS; cat. no. A3382101; Thermo Fisher, Waltham, MA). ADRB2 knockdown was performed as previously described [14], and the stably transfected cell lines LNCaP shADRB2-1, LNCaP shADRB2-2 and LNCaP shADRB2-3 were maintained in RPMI 1640 with 10% FCS supplemented with 200 μg/ml G418 sulphate (cat. no. 10131–027; Thermo Fisher, Waltham, MA). ADRB2 was overexpressed as previously described [14]. Briefly, LNCaP shADRB2-2 cells were transfected for 48h using XtremeGene HP DNA transfection reagent (cat. no. 06366236001; Sigma, St. Louis, MO) under androgen-proficient conditions with a pcDNA3 plasmid containing the full length ADRB2 gene (p-ADRB2) (cat. no. 14697; Addgene, Watertown, MA). As a control, LNCaP shADRB2-2 cells were transfected with a non-expressing/empty pcDNA3.1/Zeo vector (p-Empty).

To knockdown miR-196a, LNCaP shADRB2-2 cells were transfected with 25 nM (final concentration) of anti-miR-196a miRNA inhibitor (cat. no. AM17000; Ambion-Thermo Fisher, Waltham, MA) with Lipofectamine RNAiMAX (cat. no. 13778150; Thermo Fisher, Waltham, MA). As a control, LNCaP shADRB2-2 cells were transfected with 25 nM of anti-miR miRNA negative control (cat. no. AM17010; Ambion-Thermo Fisher, Waltham, MA).

For the β-blocker experiment, cells were stimulated with 10 μM of either ICI 118 551 (cat.no I127-5MG; Sigma, St. Louis, MO), CGP-20712 (cat.no. C231-10MG; Sigma, St. Louis, MO), SR59230A (cat.no. S8688-5MG; Sigma, St. Louis, MO), propranolol (cat.no. P8688; Sigma, St. Louis, MO), timolol (cat.no. T6394; Sigma, St. Louis, MO) or vehicle for 48 hours, followed by RNA extraction. Total RNA was extracted using TRIzol reagent from Invitrogen and following the manufactures protocol.

### Neurite outgrowth quantification

Images of LNCaP shADRB2-2 negative control and anti-miR-196a cells cultured in RPMI 1640 with 10% CSS for three days were acquired in an IncuCyte S3 Live-Cell Analysis System equipped with a 10X objective. Neurites were quantified manually in 20 cells of 10 fields (200 cells per condition). Specifically, protrusions longer than 1.5-fold the cell body were counted. Fields with similar cell aggregation and confluency were chosen. The experiment was performed three times and the images were analyzed first by one person and then blindly by a second person.

### RNA extraction and quantitative real time qRT-PCR

RNA was extracted from prostate cancer cell lines using miRNeasy Plus Kit (cat. no. 74134; Qiagen; Germany) following the instructions of total RNA extraction containing miRNA. For miRNA quantification, 100 ng of RNA were used for cDNA synthesis with the miRCURY LNA RT Kit (cat. no. 339340; Qiagen), and qRT-PCR was performed using miRCURY LNA SYBR GREEN PCR Kit (cat. no. 339346; Qiagen). miR-196a (cat. no. YP00204386; Qiagen) was analyzed using miR-16-5p (cat. no. YP00205702; Qiagen) as reference gene [28]. For mRNA quantification, 100 ng of RNA were used in the qScript One-Step qRT-PCR Kit (Quanta bio; Beverly, MA), according to the manufacturer’s protocol. DNA-directed RNA polymerase II subunit RPB1 (POLR2A) and 5-aminolevulinate synthase (ALAS1) mRNA expression were used as reference genes [14]. Both for miRNA and mRNA, the ΔΔCt method was employed to evaluate relative gene expression. The experiments were done in triplicates.

Primer sequences: ADRB2 forward: GTCTTGAGGGCTTTGTGCTC, reverse: GGCAGCTCCAGAAGATTGAC; ALAS1 forward: CTGCAAAGATCTGACCCCTC, reverse: CCTCATCCACGAAGGTGATT; KLHL1 forward: CTGAGCCAAAGATGCAGTGT, reverse: AGTTTGAAAAATGGGCGATG; NSE forward: ACTTTGTCAGGGACTATCCTGTG, reverse: TCCCTACATTGGCTGTGAACT; POLR2A forward: GCACCACGTCCAATGACAT, reverse: GTGCGGCTGCTTCCATAA.

### Immunoblotting

Cells were washed with phosphate-buffered saline (PBS) and lysed in lysis buffer (50 mM Tris-HCl, 300 mM NaCl, 1 mM EDTA, 0.5% Triton X-100, pH 7.4). Cell lysates were mixed with loading buffer, separated by 4–20% SDS-PAGE and transferred to polyvinylidene fluoride membranes. The membranes were blocked in 5% milk in PBS-Tween for 60 min, followed by overnight incubation with the following primary antibodies in 5% BSA in PBS-Tween: mouse anti-tubulin, beta 3 class III (TUBB3) (cat. no. 801201; BioLegend, San Diego, CA, USA), rabbit anti-Kelch Like Family Member 1 (KLHL1) (cat. no. ab154704; Abcam, Cambridge, UK), rabbit anti-neuron-specific enolase (NSE) (cat. no. PC237; Oncogene Research Products, San Diego, CA) and rabbit anti-p27^kip1^ (cat. no. 3686T; Cell Signaling, Danvers, MA, USA) and actin (cat. no. 177-CLT9001; Cederlane, Burlington, Canada). The antibodies were used at 1:500 to 1:1000 dilution. Then the membranes were incubated with horseradish-conjugated secondary antibodies (Jackson Immunoresearch, West Grove, PA, USA) and the signals were detected with SuperSignal West Dura Extended Duration Substrate (Thermo Fisher Scientific, Waltham, Massachusetts, USA) in a ChemiDoc Imaging System (Bio-Rad, Hercules, California, USA).

### Statistical analysis

Continuous variables were analyzed by Student’s t-test using Graphpad Prism 8. For the analysis of miRNA expression in PCa tissues in publicly available datasets, a one-way ANOVA analysis between the groups and a post hoc multiple comparison test using Tukey were performed. For all the statistical analyses, significance was set at p ≤ 0.05 (*), p ≤ 0.01 (**), p ≤ 0.001 (***) and p ≤ 0.0001 (****), respectively.

## Results

### miR-196a and ADRB2 correlation in LNCaP prostate cancer cells

**Fig 1:**
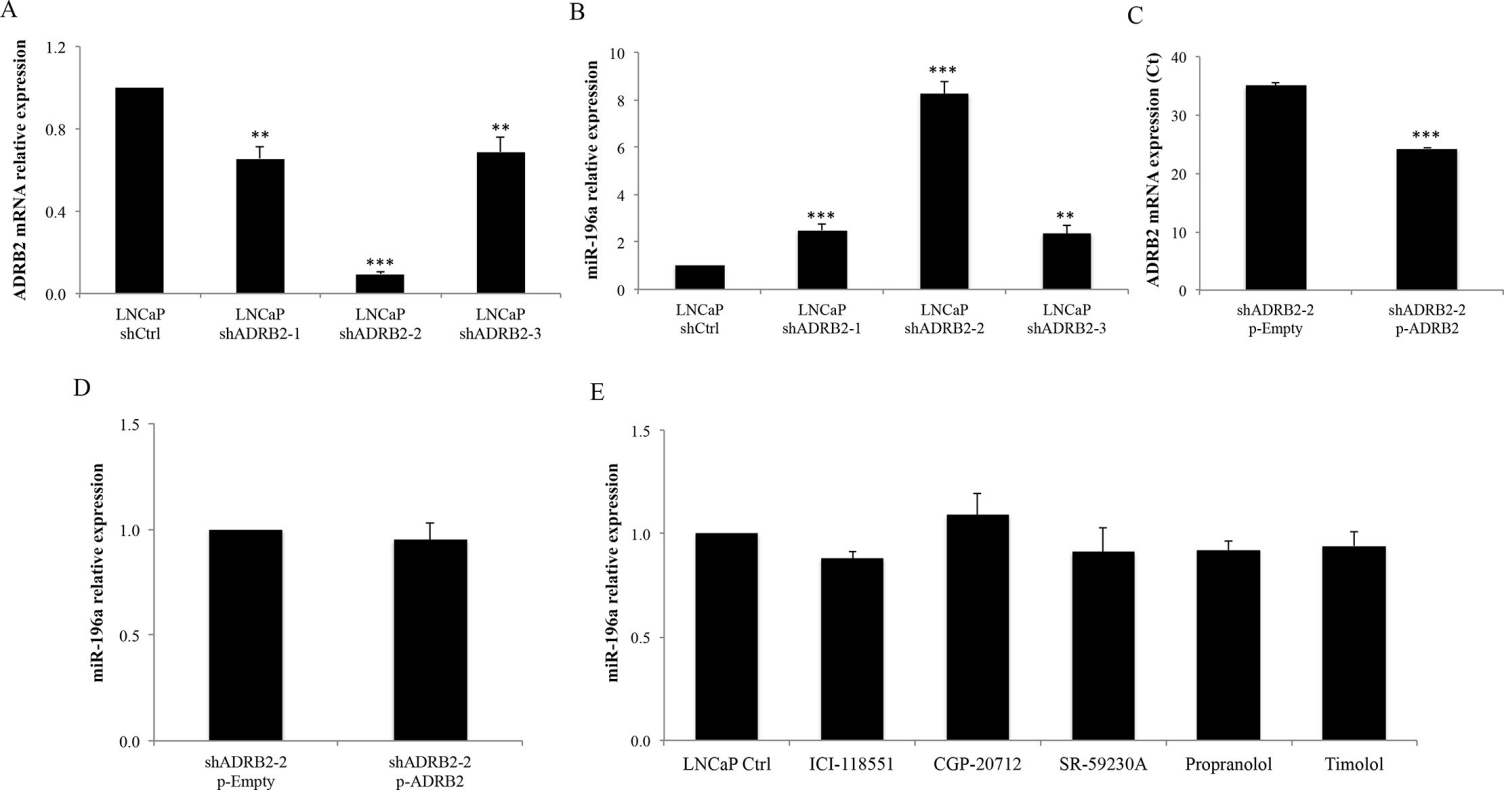
miR-196a expression in LNCaP cells with altered levels or signaling of ADRB2. (A) qRT-PCR analysis of ADRB2 in stably transfected LNCaP cells, shADRB2-1, shADRB2-2 and shADRB2-3, and negative control cells, shCtrl. A representative experiment with triplicates is shown. (B) qRT-PCR analysis of miR-196a in stably transfected LNCaP cells, shADRB2-1, shADRB2-2 and shADRB2-3, and negative control cells, shCtrl. (C) qRT-PCR analysis of ADRB2 in shADRB2-2 cells transfected with a plasmid overexpressing ADRB2 (shADRB2-2 p-ADRB2) and negative control cells (shADRB2-2 p-Empty). (D) qRT-PCR analysis of miR-196a in shADRB2-2 cells transfected with a plasmid overexpressing ADRB2 (shADRB2-2 p-ADRB2) and negative control cells (shADRB2-2 p-Empty). (E) qRT-PCR analysis of miR-196a in LNCaP Ctrl cells treated with different β-blockers or vehicle for 48 hours. The experiments were performed three times and a representative experiment showing the mean ± standard deviation of three replicates is presented in the figure. miR-196a expression was normalized to the reference gene miR-16-5p. The ADRB2 mRNA levels were normalized to the reference gene ALAS1.

The potential correlation between miR-196a and ADRB2 in prostate cancer cells was investigated in the hormone-sensitive human prostate adenocarcinoma LNCaP cell line stably transfected with shRNAs to knockdown ADRB2 expression. In particular, three stable cell lines derived from LNCaP cells previously established by our group were used: LNCaP shADRB2-1, LNCaP shADRB2-2 and LNCaP shADRB2-3. These cells lines show respectively 50%, 85% and 80% reduced ADRB2 ligand binding activity compared to control (Ctrl) LNCaP cells (LNCaP shCtrl) [8, 14]. We also showed by PCR the reduction of the mRNA levels of ADRB2 in LNCaP shADRB2-1, LNCaP shADRB2-2 and LNCaP shADRB2-3 compared to LNCaP shCtrl (Fig 1A). In this case the mRNA levels of ADRB2 were reduced by approximately 85% (6.7 times) in LNCaP shADRB2-2, and by approximately 40% (1.7 times) in LNCaP shADRB2-1 and LNCaP shADRB2-3. To study whether the levels of miR-196a were altered in cells with reduced levels of ADRB2, the amount of this miRNA was measured by qRT-PCR in the three ADRB2 knockdown cells and in control cells. As shown in Fig 1B, the level of miR-196a was upregulated in cells with reduced levels of ADRB2. Moreover, the upregulation was highest in LNCaP shADRB2-2, the cell line containing the lowest levels of ADRB2. To investigate whether the upregulation of miR-196a was directly associated to the expression of ADRB2, the levels of the receptor were rescued in shADRB2-2 cells by transient transfection with a plasmid overexpressing ADRB2 (p-ADRB2). qRT-PCR experiments showed a difference of 10 cycles between the two conditions, indicating that the levels of ADRB2 had been recovered (Fig 1C). As shown in Fig 1D, the expression of miR-196a in rescued shADRB2-2 p-ADRB2 cells was similar to control shADRB2-2 p-Empty cells. This suggests that the expression of miR-196a is increased only as a consequence of ADRB2 reduction. Furthermore, to assess if the levels of miR-196a were affected by inhibiting the signaling activity of ADRB2, LNCaP cells were treated with different β-blockers. The following compounds were used: ICI-118551, a highly selective ADRB2 inverse agonist, CGP-20712, a selective β1-adrenergic receptor antagonist and SR-59230A, a selective β3-adrenergic receptor antagonist. In addition, propranolol and timolol, two non-selective ADRB2 antagonists were used. As shown in Fig 1E, the expression of miR-196a was not affected to a large extent by any of the β-blockers, thus suggesting that the increase in the expression of miR-196a is related to the low expression of ADRB2 and not to the inhibition of the receptor signaling activity.

### miR-196a expression is upregulated in metastatic PCa tissue samples

**Fig 2:**
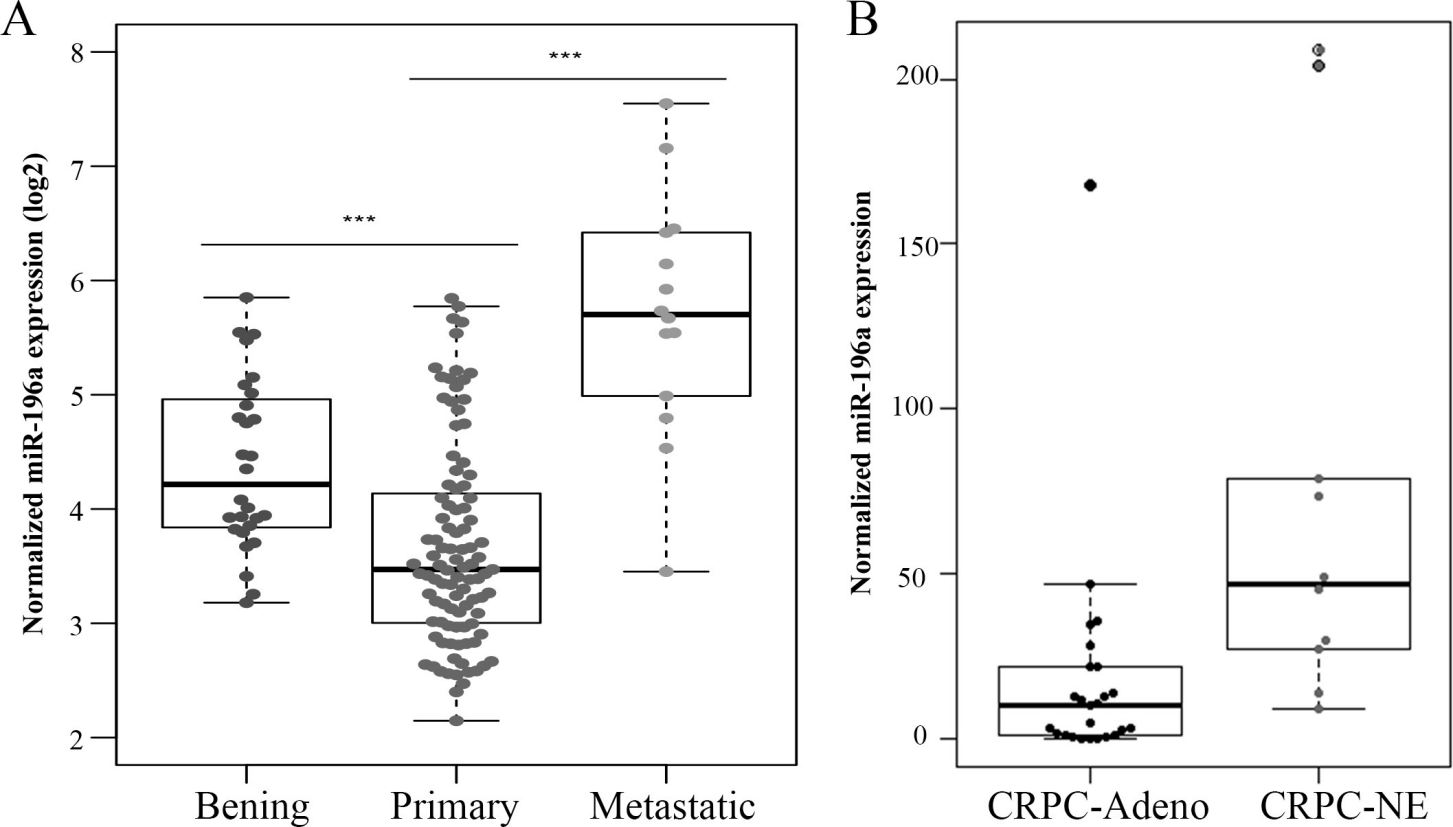
Boxplot graph of miR-196a expression in PCa samples. The publicly available dataset of prostate tissue samples, GSE21036, of miRNA expression in PCa tissues (A) and results recently published by Bhagirath *et* al. [29] (B) were used to generate these figures. A one-way ANOVA analysis between the groups and a post hoc multiple comparison test using Tukey were performed. Significance was set at p ≤ 0.05 (*), p ≤ 0.01 (**) and p ≤ 0.001 (***). CRPC-ADENO: Cohort of human metastatic castration-resistant prostate cancer (CRPC) clinical samples with no evidence of NED and patient-derived xenografts models with adenocarcinoma characteristics. CRPC-NE: Clinical samples from treatment-induced neuroendocrine (NE) prostate cancer and models of neuroendocrine prostate cancer.

We and others have previously shown that low ADRB2 expression levels are associated with short time to biochemical recurrence and increased cellular metastatic potential than high ADRB2 [14, 17]. Interestingly, a recent study showed that high expression of miR-196a was associated with worse BCR-free survival [26]. In this study, we have investigated the levels of miR-196a in metastatic PCa clinical specimens using a publicly available dataset (GSE21036). Three different groups of human prostate tissue samples were considered: primary PCa (Primary), metastatic PCa (Metastatic) and normal benign prostate (Benign). As shown in Fig 2A, miR-196a is downregulated in primary PCa tissues compared to benign samples. In addition, miR-196a is upregulated in metastatic PCa tissues compared to benign samples (Fig 2A). These data support the possible involvement of miR-196a in a metastatic cellular phenotype. Moreover, whereas low level of ADRB2 has been observed in neuroendocrine prostate cancer [14], RNA sequencing of miRNAs showed that the level of miR-196a was higher in clinical samples from treatment-induced neuroendocrine prostate cancer than in human metastatic castration-resistant prostate cancer with no evidence of NED (Fig 2B) [29].

### miR-196a inhibition increases neurite outgrowth in LNCaP cells

**Fig 3:**
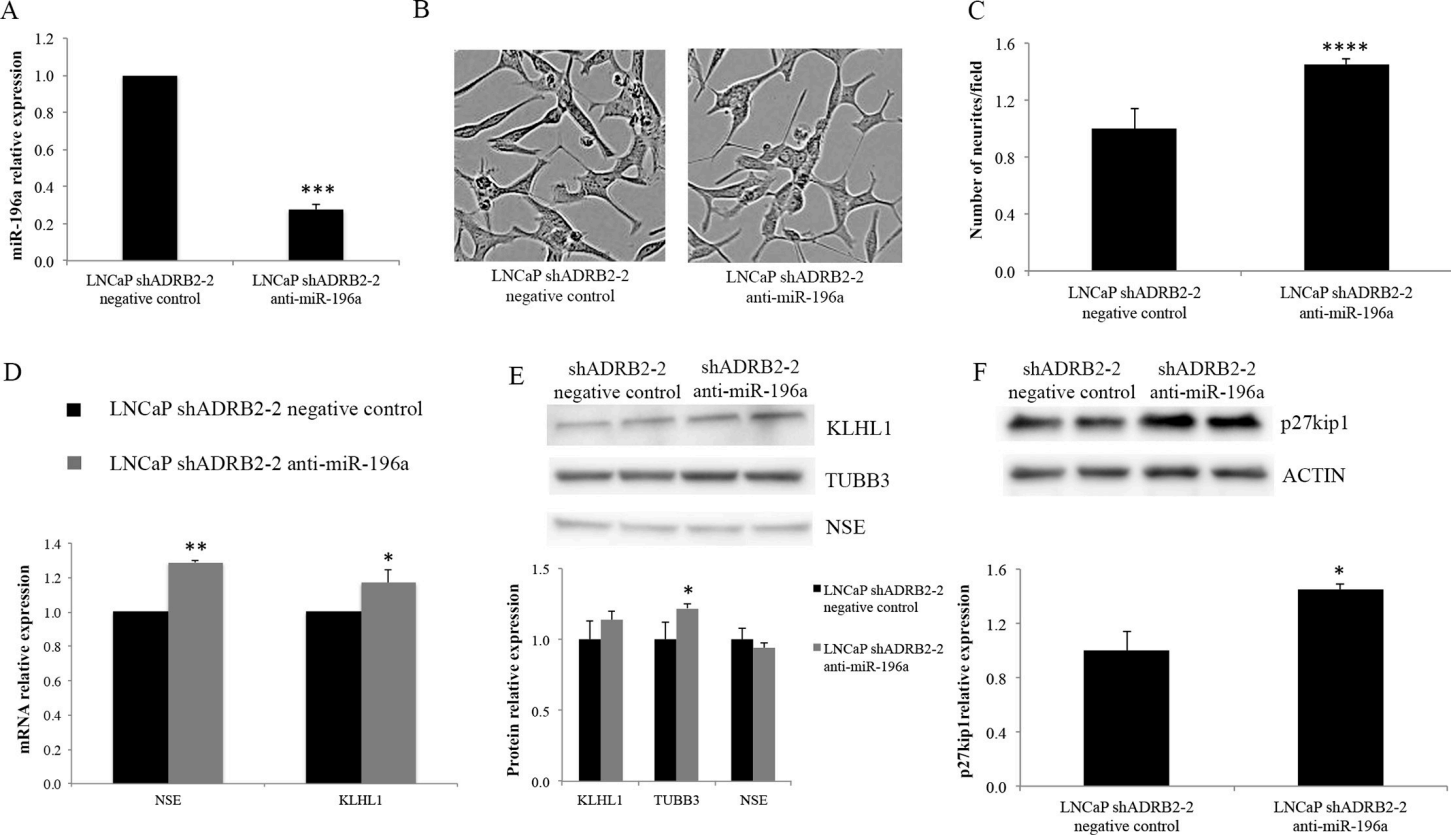
miR-196a effect in neurite outgrowth. (A) qRT-PCR analysis of miR-196a in LNCaP shADRB2-2 cells transfected with anti-miR-196a and anti-miR negative control miRNA. miR-196a expression was evaluated after 3 days in 10% CSS medium and normalized to the reference gene miR-16-5p. n = 4. (B) Representative fields from phase-contrast light microscopy images of LNCaP shADRB2-2 negative control and anti-miR-196a cells cultured in 10% CSS for 3 days. (C) Quantification of neurite-like outgrowths in LNCaP shADRB2-2 negative control and anti-miR-196a cells cultured in 10% CSS for 3 days. Bars represent the average number ±SD of neurite-like outgrowths per field. p <0.0001. Neurite-like outgrowths were quantified manually in 20 cells of 10 fields (200 cells per condition) in three different experiments. (D) qRT-PCR analysis of NSE and KLHL1 mRNA levels in LNCaP shADRB2-2 cells transfected with anti-miR-196a and anti-miR negative control miRNA. mRNA levels were evaluated after 3 days in 10% CSS medium and normalized to the reference gene POLR2A. The experiment was performed two times and a representative experiment showing the mean ± SD of three replicates is presented. (E) Western blot analysis of neuroendrocrine markers in LNCaP shADRB2-2 negative control and anti-miR-196a cells. Quantification of three experiments with duplicates and representative gels are shown. (F) Western blot analysis of p27^kip1^ in LNCaP shADRB2-2 negative control and anti-miR-196a cells. Quantification of three experiments with duplicates and a representative gel are shown.

We have recently reported that ADRB2 is involved in androgen depletion-mediated neuroendocrine trans-differentiation [14]. In that study, LNCaP cells were grown in medium with CSS deprived of steroid hormones to mimic androgen deprivation. The results showed that cells with low levels of ADRB2 had lower expression of neuroendocrine markers, and shorter as well as fewer neurite-like outgrowths than cells with high levels of ADRB2 [14]. To investigate if depletion of miR-196a could rescue neuroendocrine trans-differentiation in LNCaP shADRB2-2 cells, the cell line containing the lowest levels of ADRB2 (Fig 1A) was transiently transfected with anti-miR-196a. In these experiments, cells were cultured for three days in medium with 10% CSS to induce trans-differentiation. Control experiments showed that the levels of miR-196a were reduced by approximately 70% (3.3 times) in cells treated with anti-miR-196a compared to cells treated with a non-targeting miRNA (Fig 3A). Both control cells and miR-196a depleted cells contain several cells with neurite-like outgrowths (Fig 3B). In each experiment, the number of neurite-like outgrowths in 20 cells of 10 fields with similar cell aggregation and confluency was quantified. As shown in Fig 3C, cells where miR-196a had been depleted had approximately 1.5-fold more neurite-like outgrowths compared to control cells. This indicates that the low capacity of cells with reduced levels of ADRB2 to undergo neuroendocrine trans-differentiation in androgen depletion conditions is mediated to some extent by miR-196a. In order to investigate whether this effect was associated to changes in the expression of neuroendocrine markers, the mRNA levels of NSE and KLHL1 were investigated by qRT-PCR. NSE is a glycolytic enzyme specific of neurons and peripheral neuroendocrine cells [30], and KLHL1 is an actin-binding protein involved in the maintenance of the ordered cytoskeleton and in the regulation of neurite outgrowth [31]. After knocking down miR-196a expression in LNCaP shADRB2-2 cells, a trend of increase of NSE and KLHL1 mRNA levels was observed (Fig 3D). We also performed Western blot to detect the protein levels of NSE and KLHL1, and of an additional neuroendocrine marker, TUBB3. As shown in Fig 3E, the levels of TUBB3 and KLHL1 were slightly increased. Finally, little is known about miR-196a and prostate cancer and the targets that it regulates but, interestingly, it has recently been shown that p27^kip1^ is a target of miR-196a in LNCaP cells [26], and we have confirmed this result by Western blot (Fig 3F). This opens the possibility for the potential implication p27^kip1^ in the observed effects of miR-196a.

## Discussion

miR-196a has been implicated in several cancers [22–24], but its role in PCa has not received much attention. In this study we have found a correlation between the levels of miR-196a and ADRB2 in LNCaP cells. Our results show that miR-196a expression is inversely correlated to ADRB2 levels in these cells. Moreover, it has previously been shown that ADRB2 functions as a molecular switch for NED in PCa, and that the level of ADRB2 is progressively reduced during tumor dedifferentiation [14]. We show here that miR-196a is implicated in this effect of ADRB2 since low ADRB2 LNCaP expressing cells acquire a more NED phenotype after anti-miR-196a transfection. Interestingly, miR-196a has recently been shown to be upregulated in samples representing neuroendocrine prostate cancer compared to castration-resistant prostate cancer with adenocarcinoma characteristics [29].

Concerning the levels of miR-196a in PCa tissue samples, we have observed some discrepancies when different cohorts of samples are used. In 2008 Ambs *et al*. analyzed 60 PCa samples and showed that miR-196a expression was increased in PCa samples compared to non-tumor tissues [25] and in a recent study Zhan *et al*. showed a similar result in 16 paired tumor and non-tumor samples [26]. In this study we used a publicly available dataset of miRNA expression in 208 tumor samples (GSE21036), including primary and metastatic PCa. We found that miR-196a expression was increased in metastatic PCa tissues and reduced in primary PCa tissues compared to benign samples. We also observed a reduction of miR-196a in primary PCa compared to benign tissue in the prostate cancer cohort from The Cancer Genome Atlas program (TCGA-PRAD) (n = 543). It is plausible that the different miR-196a expression results are due to their different clinical characteristics and/or sample size. Interestingly, as it is the case for ADRB2, miR196a expression also changes during PCa progression. It should also be mentioned that miR-196a is downregulated in urinary extracellular vesicles of PCa patients compared to healthy controls [27], and that this may be due to the lower levels of miR196a in primary prostate cancer.

Our results show that the inhibition of miR-196a leads to a NED phenotype in LNCaP cells expressing low levels of ADRB2. LNCaP cells are often used to study NED, but it would be interesting to investigate whether a similar effect is observed in other prostate cancer cell lines such as PC-3, C4-2B or VCaP. It can be expected that in these cells, similarly to LNCaP, the effect depends of the depletion extent of the ADRB2 levels. However, the effect observed in LNCaP cells seems to differ from observations in mouse neuroblastoma N2a cells and primary neurons. In these cells miR196a has been shown to induce neurite outgrowth [32, 33], and in N2 cells the effect of miR196a was mediated by the suppression of the ran-binding protein 10 gene [33]. Neurite outgrowth was also increased by miR196a in N2a cells mimicking Huntington’s disease conditions [32]. Interestingly, this neuroprotective effect of miR196a opens for a potential therapeutic use of this miRNA in the disease. In fact, miR-196a delivery has been shown to have a positive effect in another neurodegenerative disease, spinal and bulbar muscular atrophy, in a mouse model [34]. The divergent effects of miR-196a in different systems are probably explained by different genes being targeted. It should be mentioned that ADRB2 is neither a validated nor a predicted target of this miRNA. However, p27^kip1^ has recently been shown to be a target of miR-196a and to mediate the effect of miR-196a in prostate cancer proliferation [26]. Interestingly, p27^kip1^ has been shown to inhibit RhoA, and RhoA inhibition is known to induce neurite outgrowth [17, 35]. In conclusion, our results propose a potential role of miR-196a in NED in an *in vitro* model of PCa. Further experiments are required to obtain additional mechanistic information and explore the therapeutic possibilities of miR-196a.

## Supporting information

S1 Raw images (PDF)
