# Supplementary material for: Implication of β2-adrenergic receptor and miR-196a correlation in neurite outgrowth of LNCaP prostate cancer cells
Source: PLoS One. 2021 Jun 30;16(6):e0253828. doi: 10.1371/journal.pone.0253828 (PMC8244869; doi:10.1371/journal.pone.0253828)
Supplement: S1 Raw images — (PDF) [file pone.0253828.s001.pdf]

Fig.3E

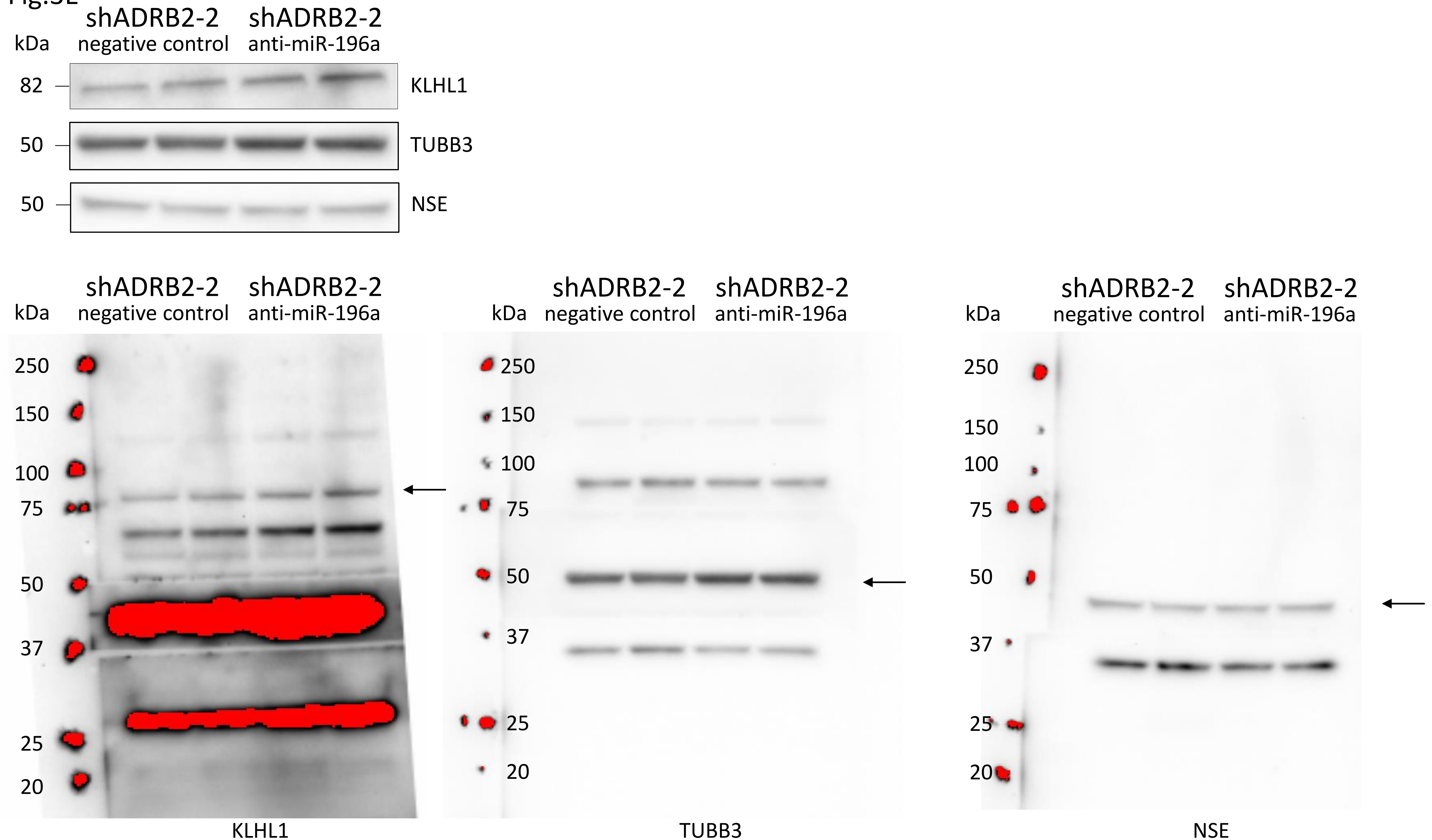
shADRB2-2 negative control
shADRB2-2 anti-miR-196a
kDa
82
KLHL1
50
TUBB3
50
NSE
250
150
100
75
50
37
25
20
KLHL1
TUBB3
NSE

Fig.3F

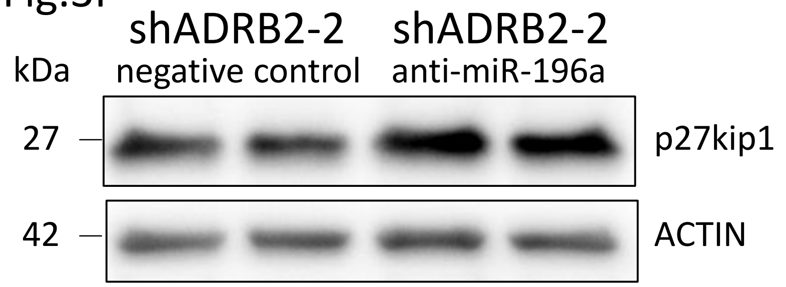
shADRB2-2
negative control
shADRB2-2
anti-miR-196a
kDa
27
42
p27kip1
ACTIN

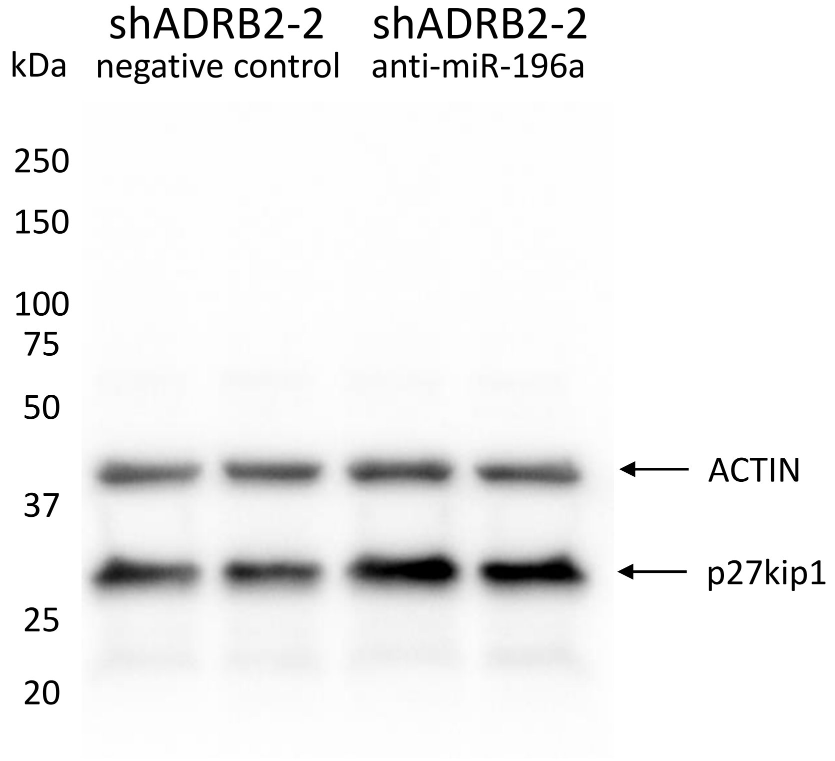
shADRB2-2
negative control
shADRB2-2
anti-miR-196a
kDa
250
150
100
75
50
37
25
20
ACTIN
p27kip1
